# Diagnostic Value and Interreader Agreement of the Pancreaticolienal Gap in Pancreatic Cancer on MDCT

**DOI:** 10.1371/journal.pone.0166003

**Published:** 2016-11-28

**Authors:** Khoschy Schawkat, Wolfgang Kühn, Daniel Inderbitzin, Beat Gloor, Johannes T. Heverhagen, Val Murray Runge, Andreas Christe

**Affiliations:** 1 Department of Diagnostic, Interventional and Pediatric Radiology, Inselspital, University Hospital Bern, Bern, Switzerland; 2 Department of Visceral and Transplantion Surgery, Inselspital, University Hospital Bern, Bern, Switzerland; 3 Department of Surgery, Tiefenau Hospital, Bern, Switzerland; 4 Department of Radiology, Tiefenau Hospital, Bern, Switzerland; Chang Gung Memorial Hospital Kaohsiung Branch, TAIWAN

## Abstract

**Objective:**

The aim of this retrospective study was to evaluate the diagnostic value and measure interreader agreement of the pancreaticolienal gap (PLG) in the assessment of imaging features of pancreatic carcinoma (PC) on contrast-enhanced multi-detector computed tomography (CE-MDCT).

**Materials and Methods:**

CE-MDCT studies in the portal venous phase were retrospectively reviewed for 66 patients with PC. The age- and gender-matched control group comprised 103 healthy individuals. Three radiologists with different levels of experience independently measured the PLG (the minimum distance of the pancreatic tail to the nearest border of the spleen) in the axial plane. The interreader agreement of the PLG and the receiver operating characteristic (ROC) curve was used to calculate the accuracy of the technique.

**Results:**

While the control group (n = 103) showed a median PLG of 3 mm (Range: 0 – 39mm) the PC patients had a significantly larger PLG of 15mm (Range: 0 – 53mm)(p < 0.0001). A ROC curve demonstrated a cutoff-value of >12 mm for PC, with a sensitivity of 58.2% (95% CI = 45.5–70.1), specificity of 84.0% (95% CI = 75.6–90.4) and an area under the ROC curve of 0.714 (95% CI = 0.641 to 0.780). The mean interreader agreement showed correlation coefficient r of 0.9159. The extent of the PLG did not correlate with tumor stage but did correlate with pancreatic density (fatty involution) and age, the density decreased by 4.1 HU and the PLG increased by 0.8 mm within every 10 y.

**Conclusion:**

The significant interreader agreement supports the use of the PLG as a characterizing feature of pancreatic cancer independent of the tumor stage on an axial plane. The increase in the PLG with age may represent physiological atrophy of the pancreatic tail.

## Introduction

Pancreatic carcinoma (PC), which is well known as a highly lethal malignancy and a recalcitrant cancer, has a 5-y survival rate of 6% [1, 2]. This poor prognosis is mainly due to late clinical presentation. Studies investigating the timeline of PC progression have shown that the onset of symptoms in PC coincides with surgical unresectability [3, 4]. The only potential curative therapy for PC at this time is complete surgical resection, but only 20% of PC cases are eligible for resection at the time of diagnosis. There is an urgent need for early detection because the number of new PC cases in the United States is estimated to double (from 43,000 to 88,000) between 2010 and 2030 [5].

Early detection screening, which utilizes inexpensive and accurate diagnostic tests, is an effective strategy for improving the survival rate. Recent studies have identified several promising biomarkers from saliva, blood, stools and pancreatic juice [1]. However, tests that are sensitive or specific enough for clinical use do not yet exist [6].

Multi-detector computed tomography (MDCT) plays a pivotal role in both the initial diagnosis and the appropriate staging of PC. The key to influencing prognosis through imaging studies is the detection of early stage lesions. It has been shown that tumor size at diagnosis is the single most important prognostic indicator in PC [4, 7, 8]. While the progression of pancreatic intraepithelial neoplasia (PanIN), a precursor lesion of PC, to an invasive tumor occurs in minute lesions (< 10 mm; 5–8 mm), extrapancreatic metastases are observed in the majority of patients with lesions ≥ 1 cm [1, 9]. Even MDCT, with its high special resolution, can only detect PCs that are ≥ 1 cm [1, 10]. Because MDCT lacks the sensitivity to detect small lesions, 90% of PC cases are diagnosed when the lesions are large (>2 cm), and often by then, they have local extrapancreatic spread [11]. Among PC cases, 10 to 15% are isodense and therefore occult on MDCT [12, 13]. Therefore, identifying noninvasive imaging signs that raise suspicion of a pancreatic malignancy prior to a visible mass formation is required.

On occasion, even for larger pancreatic lesions, the diagnosis of a pancreatic head tumor, which accounts for 75% of all pancreatic cancers, is difficult to differentiate from focal or chronic pancreatitis or metastatic disease. No single imaging criterion can perform this differential diagnosis with certainty. Malignant imaging features strongly indicating a neoplastic disease of the pancreatic head, such as a double-duct sign or upstream main pancreatic duct dilatation, may be observed in cases of focal pancreatitis. Other authors have conducted large surgical studies to show that in 5% of cases, only pancreatitis is found in the resected specimens that are suspicious for pancreatic head tumors [14]. Further secondary signs of a pancreatic mass, such as fullness of the pancreatic head by loss of the lobular appearance of the pancreatic parenchyma, contour abnormalities and pancreatic tail atrophy, are considered to be indicators of malignancy even if no elusive mass is detected.

Several studies have demonstrated that surgical ligation of the pancreatic duct leads to significant pancreatic atrophy [15–18].

We focused on malignant imaging features on MDCT with an emphasis on pancreatic tail atrophy leading to a PLG through atrophy of the pancreatic tissue in its longitudinal dimension. Therefore, the purpose of this retrospective study was to assess the PLG, a potential malignant imaging feature of PC, on cross-sectional imaging. In addition, we investigated the age-dependent shrinking of the pancreas, the fatty atrophy, the interreader agreement of PLG among radiologists, and the correlation between the PLG and tumor stage.

## Materials and Methods

### Patients

Institutional Review Board (IRB) was waived (University hospital Bern) due to the retrospective nature of this study. De-identified anonymised patient data were accessed for this study during a time period of three month (June to August 2015). The inclusion criteria were as follows: Preoperative contrast-enhanced multi-detector computed tomography (CE-MDCT) in the portal venous phase and postoperative confirmation of PC with tumor staging according to the World Health Organization (WHO) criteria or follow-up/tumor monitoring for patients with unresectable, histologically proven pancreatic cancer. Patients with splenic alterations, such as splenomegaly, splenectomy or continuous metastatic disease from the pancreatic tail to the spleen, were excluded. Patient with pancreatic diseases, such as parenchymal calcifications post pancreatitis, intraductal concrement in the main pancreatic duct and intraparenchymal cystic lesions, were also excluded.

The initial database search yielded 98 patients, 8 of whom were excluded because of a missing presurgical CT. Of the remaining 90 patients, 22 without PC were excluded because the initial diagnosis of pancreatic cancer was not confirmed by a pathological evaluation or the tumor origin remained unclear (18 patients), cholangiocarcinoma was initially misinterpreted as pancreatic cancer on diagnostic imaging (2 patients), or pancreatitis was present (2 patients). Of the remaining 68 patients with PC, two were excluded due to metastastic disease of the spleen by direct tumor invasion. The final cohort included 66 patients. The control group consisted of 103 age- and gender-matched healthy individuals who were recruited randomly from a population with normal abdominal CTs.

To assess the age-dependent shrinking of the pancreas, we investigated the PLG of 20 healthy term newborn infants on ultrasound to assess the starting point. To analyze the age-related development of the PLG in the control groups (0–40 y), we investigated the PLG on CT scans of always 10 individuals with normal pancreases and spleens in the age groups of 0–10 y, 11–20 y, 21–30 y and 31–40 y.

In order to quantify the fatty atrophy of the pancreas the density of the pancreatic tail was measured in Houndsfield-Units (HU) on non-contrast native axial CT-Images of 1mm slice thickness. Lower densities representing fattier parenchyma. A ROI was set inside the boarder of the pancreatic tail (>5mm diameter) avoiding inclusion of widened pancreatic ducts and cysts. Patients with pancreatic carcinoma located in the pancreatic tail were excluded. Also, patients with only post contrast images were excluded, due to the serious confounding factor of the different individual and phase-dependent enhancement. A total of 47 patients could be identified with additional non-contrast images in the carcinoma group (23 patients) and in the control group (24 patients). Age-matching was performed with averaging the densities per decennial groups. In addition 14 native CT were found and evaluated in the age group below 40 years with normal pancreases to analyze the aging process as continuous spectrum between 0 and 90 Years. Patients with age lower than one year old were grouped into the age group 0 years.

### Measurement technique

In a pilot study of PLG measurements we examined the differences of axial and coronal measurements. We could demonstrate that the axial measurement is closer to the true 3D reformatted closest gap between pancreas and spleen (real truth in 3D mode, syngo-via, siemens germany, reformatted plane along the axis of the pancreatic tail):

PLG measured on axial plane and PLG measured on 3D reformatted plane along the pancreatic tail shows significant better correlation coefficient r = 0.901 (95% CI for r = 0.801 to 0.952) compared to PLG measured on coronal plan vs. PLG on reformatted plan with a correlation coefficient r = 0.670 (95%CI for r = 0.408 to 0.830) with a p = 0.0151 (z-statistic comparison of correlation coefficient).

PLG measured on axial plane is an approximated value with an absolute error of 0.95mm +/- 1.8mm (mean +/- SD) compared to the true reformatted closest gap between pancreas and spleen on 3D reformatted plane. Measurements of the PLG on coronar plane showed a significant higher absolute error of 6.8 mm +/-10.0mm (mean +/- SD) with a p-value of 0.0093 (Wilcoxon paired rank sum test).

There was no significant difference between the interreader agreement of the PLG measurements conducted on axial plane (correlation coefficient r = 0.8641) compered to those on coronal plane (correlation coefficient r = 0.8670) with a p-value of 0.9731 (z-statistic comparison of correlation coefficient).

In order to provide a simple way to access the PLG on routine scans for different scan protocols and post processing procedures we suggest measuring the PLG on axial plane.

### Pathologic Analysis

After a tumorectomy, which was generally performed by pancreaticoduodenectomy (Whipple procedure) or distal pancreatectomy, the resected specimen was reviewed by a gastrointestinal pathologist. The tumor was graded according to the WHO classification on hematoxylin and eosin-stained specimens on the basis of the most aggressive features indicative of carcinoma, such as a haphazard growth pattern or perineural and/or intravascular invasion [19].

For tumor staging, both the CT scans and the pathologic assessment of the resected specimen were used to evaluate the local invasion of the tumor to the adjacent soft tissue or the vasculature. Diagnostic imaging revealed the presence of any metastasis.

### Imaging Technique

As some patients initially presented at peripheral institutions, 26 initial scans in the portal venous phase with variable slice thicknesses (1- to 5-mm slices) on the axial plane were imported and analyzed on our institution’s workstations. An additional 65 scans were performed on a dual-source MDCT scanner at our institution (Somatom Definition Flash, Siemens Healthcare, Erlangen, Germany) using our standard abdominal CE-MDCT protocol in the portal venous phase. Axial 1-mm slices and 5-mm slices were reconstructed. Patients were imaged after IV administration of 110 ml of an iodinated contrast medium (Ultravist 370, iopromid, 370 mg l/ml, Bayer Healthcare, Leverkusen, Germany) injected at 2 ml/s. The following scanning parameters were used: pitch, 1.0; rotation time, 0.5 s; 120 reference kVp and 140 reference mAs (carekV and caredose); and scanning delay, 70 s for the portal venous phase. The single remaining scan was performed on a PET/CT, and therefore, the PLG was evaluated on unenhanced 5-mm slices for this patient. All scans were reviewed on a PACS workstation (Picture Archiving and Communication System IDS7; Sectra, Linkoping, Sweden).

### Imaging Interpretation

Three radiologists, a radiologist specialized in abdominal and thoracic imaging with 16 y of experience, a fellow specializing in body imaging with 10 y of experience and a junior faculty radiologist with 2.5 y of body imaging experience, independently reviewed the scans. The PLG was assessed on the axial plane on the image that yielded the smallest distance between the very distal end of the pancreatic tail and the closest border of the spleen to the pancreatic tail. Measurements were recorded on the thinnest axial image reconstruction available, and 90% of measurements were obtained from images with slice thicknesses between 1 and 2 mm. The readers independently selected the specific slice on which the PLG showed the smallest distance.

### Statistical Analysis

We recorded patient characteristics, such as age; gender; anatomical localization of the pancreatic tumor; tumor staging according to the WHO classification (retrieved from the integrated patient dossier (i-pdos) clinical information system (KIS), CompuGroup medical (CGM), Phoenix, version 7.8.0.1.5); and the PLG. The Mann-Whitney test was used to compare the PC and control groups. Tumor staging was pooled into Stages I and II versus Stages III and IV (Mann-Whitney test). Within the control group, we analyzed the age-dependent development of the PLG. To calculate the age-dependent shrinkage, we used a linear curve fitting that assumed a PLG of 0 mm at birth (pilot study). To analyze the age dependent fatty atrophy of the pancreas a curve fitting was used on the pancreatic HU. Interreader agreement of the PLG was evaluated using a Bland-Altman plot for ordinal features. A receiver operating characteristic (ROC) curve was applied to calculate the cutoff gap for PC and the area under the curve to represent accuracy. Statistical significance was defined as p < 0.05. Statistical analyses were performed on MedCalc® Version 7.6.0.0 (MedCalc Software, Mariakerke, Belgium). In addition, the correlation between location of PC (head, body and tail) and PLG was analyzed using the Mann-Whitney test

## Results

The dataset yielded 66 patients (36 male and 30 female; median ages 66 and 65 y, respectively; range, 19–88 y).

The PC was localized in the pancreatic head in 46 patients (70%), in the pancreatic body in 8 patients (12%) and in the pancreatic tail in 12 patients (18%). The highest prevalence of PC was found in the 60–69 y age group. The majority of our cohort was diagnosed when lesions were already involved in adjacent major blood vessels (63%; Stages III and IV). Extension beyond the pancreas (Stage II-IV) was found in 97% of the patients. Only one patient (2%) was diagnosed with a precursor lesion (PanIN II) before it became invasive. The distribution of the tumor staging of the primary lesion at the initial scan is summarized in Table 1.

**Table 1:** Tumor staging of the primary lesion at the initial scan according to UICC (Union for International Cancer Control).

*Tumor staging*	*0 and Ia*	*Ib*	*IIa*	*IIb*	*III*	*IV*
N (%)	1 (2)	1 (2)	4 (6)	18 (27)	18 (27)	24 (36)
PLG mean (mm)	5	31.0	15.0	15.0	21.6	24.2
SD (+/-)	0.0	0.0	14.7	14.9	10.7	17.2

**Fig 1:**
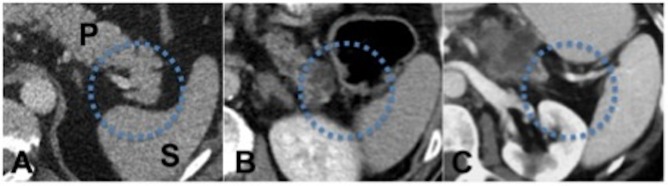
Patients with PC show a significantly larger PLG compared to the control group. (P: pancreas, S: spleen). A: Control group with normal pancreases. Patients with pancreatic head carcinoma (B) and pancreatic tail carcinoma (C) show a significantly larger PLG compared to the control group (PLG > 10 mm).

**Fig 2:**
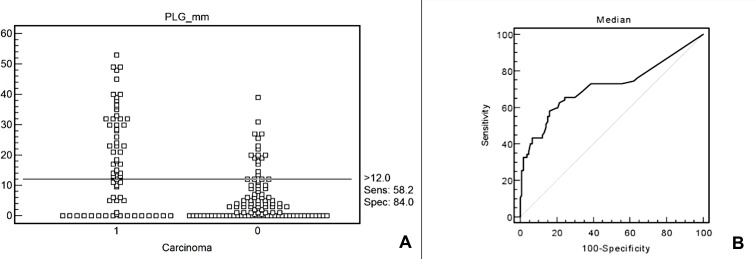
Cut-off value for carcinoma of > 12 mm; Sensitivity: 58.2 (95% CI = 45.5–70.1), Specificity: 84.0 (95% CI = 75.6–90.4) (Fig 2A). Area under the ROC curve = 0.714 (95% CI = 0.641 to 0.780) (Fig 2B).

While the control group (n = 103) showed a median PLG of 3 mm (Range: 0 – 39mm) the PC patients had a significantly larger PLG of 15mm (Range: 0 – 53mm)(p < 0.0001) (Fig 1). A ROC curve demonstrated a cutoff-value of >12 mm for PC, with a sensitivity of 58.2% (95% CI = 45.5–70.1), specificity of 84.0% (95% CI = 75.6–90.4) and an area under the ROC curve of 0.714 (95% CI = 0.641 to 0.780) (Fig 2). The mean interreader agreement showed correlation coefficient r of 0.9159 (Table 2). The extent of the PLG did not correlate with tumor stage but did correlate with the age, the PLG increased by 0.8 mm within every10 y.

**Table 2:** The mean interreader agreement for the three readers showed correlation coefficient r of 0.9159.

*Interreader correlation*	*Correlation coefficient r*	*95% Confidence interval*	*p-value*
Reader 1 –reader 2	0.8419	0.5796	0.9461
Reader 1- reader 3	0.9329	0.8057	0.9779
Reader 2 –reader 3	0.9729	0.9184	0.9912
Average	**0.9159**	**0.7679**	**0.9717**

PC located in the pancreatic body showed a larger PLG indicative for a larger degree of longitudinal atrophy (p-value: 0.0926, Table 3) compared to PC located in the pancreatic head.

**Table 3:** Correlation of PC location and PLG. Compared to PC in the pancreatic head PC located in the pancreatic body demonstrated a larger PLG indicative of a higher degree of longitudinal pancreatic atrophy.

*Tumor location*	*PLG*	*range*		*p-value*
head	16	0–48.5	head vs. body	0.0926
body	23	15–57	body vs. tail	**0.0031**
tail	1	0–38	head vs. tail	**0.0101**

**Fig 3:**
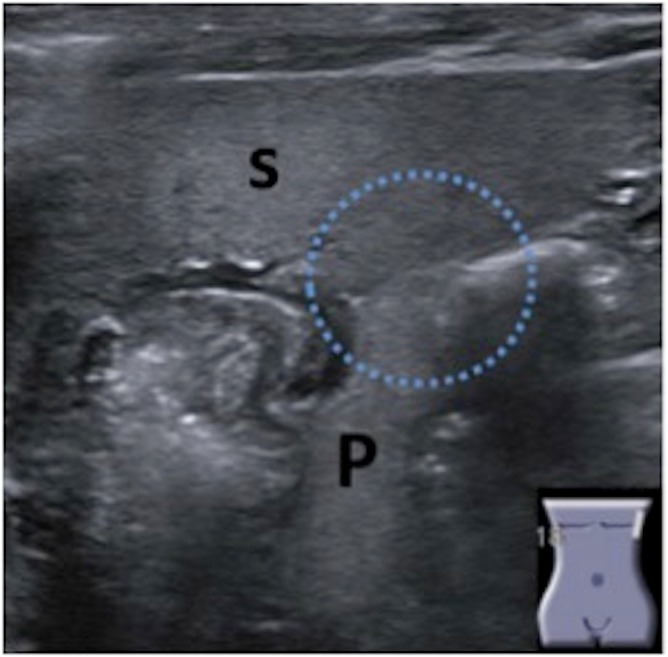
An investigation of the PLG in 20 healthy term newborn infants showed a PLG of 0 on ultrasound with direct contact of the pancreatic tail with the splenic border.

**Fig 4:**
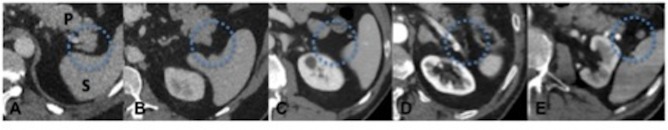
Age-related development of the PLG in the control group. The PLG is age correlated and increases by 0.8 mm within 10 years (P: pancreas, S: spleen). A: age group 40–50 y. B: age group 50–60 y, C: age group 60–70 y, D: age group 70–80 y, E: age group 80–90 y.

**Fig 5:**
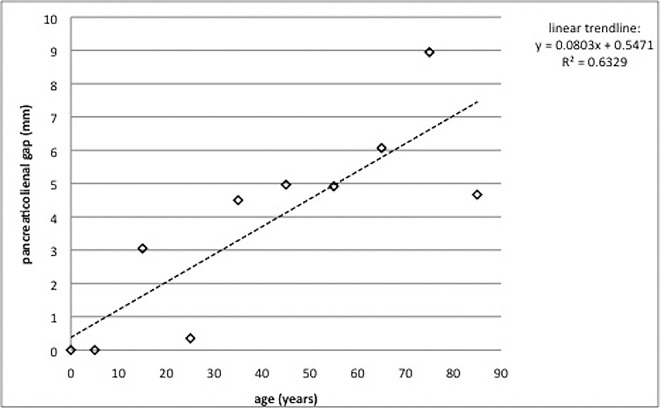
Age-related development of the PLG in the control group. Linear curve fitting demonstrated a good correlation between the PLG and age (R^2^ >0.5): The PLG increased by an average of 0.8 mm every 10 years, with a PLG of 0 mm at birth and a hypothetical gap of 8.6 mm at 100 y.

In the pilot study, the 20 healthy term newborn infants (8 male and 12 female, age: 1 to 28 d) showed a PLG of 0 (Fig 3). Within the newborn and the control groups, we analyzed the age-dependent development of the PLG. The PLG is age correlated and increases by 0.80 mm within 10 years (Figs 4 and 5).

**Fig 6:**
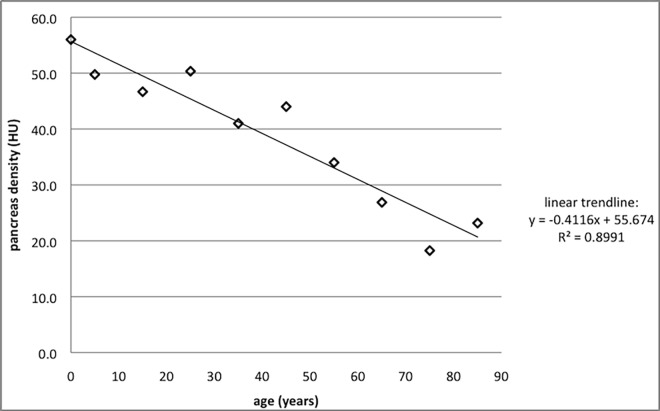
Fatty atrophy of pancreas: on average the density of the pancreas decreases by 4.1 HU per decennium.

Fatty atrophy with aging: There was no significant difference in the density of the pancreatic tissue between the carcinoma and the control group: the mean density measured 27.0 HU +/- 16.7 SD and 27.5 HU +/- 13.8 SD, respectively (p-value = 0.9). The density of the pancreas decreases with age due to fatty atrophy (Fig 6). At birth the average density equals 56.0 HU+/- 12.7 SD and decreases on average 4.1 HU per 10 years to reach a hypothetical density of 15 HU at the age of 100.

The density of the pancreas (pancreatic tail) correlates significantly to the PLG: the correlation coefficient equals -0.882 (95%CI -0.9718 to -0.5668; p-value = 0.0007).

## Discussion

Several indirect imaging features on MDCT have been associated with malignancy of the pancreas in the absence of a visualized mass, including a double-duct sign, upstream main pancreatic duct dilatation, contour abnormalities, loss of lobulation and pancreatic tail atrophy [20–22]. However, these features are difficult to quantify and lack reproducibility. To the best of our knowledge, this is the first study to quantify pancreatic tail atrophy by measuring the pancreaticolienal gap and to identify a reproducible indirect malignant feature.

Our patient cohort comprised a representative selection of patients with pancreatic carcinoma. Regarding tumor location, our results are consistent with published data [2], with the PC localized in the pancreatic head in 46 patients (70%), in the pancreatic body in 8 patients (12%) and in the pancreatic tail in 12 patients (18%).

Our results show that a PLG larger than 12 mm is associated with pancreatic carcinoma. Patients without pancreatic carcinoma had a smaller or absent PLG. The significant interreader agreement supports the use of the PLG for characterizing malignant features of pancreatic cancer on MDCT.

Different studies have investigated the double-duct sign. Sensitivities and specificities of this sign for PC range from 50–76% and from 63–80%, respectively [23–25]. A pathologic PLG (>12 mm) delivers a comparable sensitivity of 58.2% (95% CI: 45.5–70.1) and a specificity of 84.0% (95% CI: 75.6–90.4), and the confidence intervals match the range of published sensitivities and specificities of the double-duct sign. In addition, 58% of patients with a pancreatic malignancy demonstrate a double-duct sign [26]. Similarly, in our cohort, 42 of 66 patients with PC (63%) showed a pathologic PLG. The correlation between PC with a double duct and pathologic PLG is the focus of an ongoing study.

Interestingly, our results show that the PLG is age correlated and is not dependent on tumor stage. Sato et al. published a study on age-dependent atrophy that stated that atrophy is most prominent in the pancreatic tail on MR images of the normal adult pancreas. They measured the anterioposterior diameter in the head, body and tail [27]. In our study, we can confirm that the longitudinal diameter of the pancreas also decreases with age. Assuming there is no PLG at birth (pilot study of 20 healthy term newborn infants), we discovered a longitudinal atrophy of 0.8 mm in 10 y, leading to a hypothetical shrinkage of 8 mm in 100 y. A decreasing spleen volume may influence the pancreatolienal gap as well, but it is clear that the increasing gap with aging is well below the 10-mm threshold for PC. The tumor stage is not significantly correlating with the PLG. There is a tendency toward larger gaps in later stages of PC. However, smaller primaries are already able to metastasize and upgrade the tumor staging without substantially obstructing the pancreatic duct and, despite a higher staging classification, do not show an enlargement of the PLG.

Regarding the tumor location PC located in the pancreatic body showed a larger PLG indicative for a larger degree of longitudinal atrophy (p-value: 0.0926) compared to PC located in the pancreatic head. The higher degree of longitudinal pancreatic atrophy in PC of the pancreatic body compared to those of the pancreatic head could be due to upstream of pancreatic enzymes in a high concentration restricted to the pancreatic tail leading to a higher degree of atrophy.

There is significant difference between the PLG due to tumor located in the pancreatic tail compared to tumor located in the pancreatic head and body with shorter PLG for tail located tumors due to direct invasion of the splenic hilus.

Obviously, there is no additional fatty atrophy in the pancreatic tail in carcinoma patients. It seems that the replacement of pancreatic tissue exists only in aging and not in the relative fast longitudinal atrophy due to tumor-obstruction. We demonstrated a decennial decrease in HU of the pancreatic tail of 4.1 HU which is in concordance with previous published studies [28]. Saisho et al. showed that pancreatic fat increases with aging in both men and women, to a greater extent in men with the composite effects of decreased parenchymal and increased fat volume. It correlates significantly with the longitudinal atrophy of the pancreas with aging, but not with longitudinal atrophy due to pancreatic cancer.

This study was limited by its retrospective nature. A total of 26 initial scans exhibiting pancreatic tumors were performed at peripheral institutions and imported to our workstation with variable slice thicknesses (1- to 5-mm slices). As all scans performed at our institution were read at 1-mm slices, the lack of available high-quality, thin-section images from other institutions may have caused an underestimation of the PLG in these patients. In addition, the scan protocols varied among different institutions. However, this inconsistency in CT protocols provided us with the possibility of comparing the influence of the enhancement phases of pancreatic tissue and the spleen on the measurability of the PLG. It is our impression that the enhancement pattern does not influence the visibility of the splenic border and the pancreatic tail and, therefore, does not have an impact on the PLG. Further studies on this issue are needed. In addition, the nonexistent PLG at birth needs to be confirmed in a larger study population. Because of the relatively specific inclusion criteria, the possibility of a selection bias cannot be ruled out. However, we enrolled a representative selection of patients with pancreas carcinoma in this study.

In summary, patients with PC show a significantly larger PLG compared to the control group, with a cutoff of 10 mm. The significant interreader agreement supports the use of PLG for characterizing malignant features of pancreatic cancer independent of tumor stage on the axial plane. Our results indicate a promising role for the PLG as an indirect malignant feature for tumor detection, which has comparable accuracy to the double-duct sign, in patients with pancreatic tumors. A prospective study to validate the use of the PLG and its added value in tumor detection is still warranted.
